# Vibrio cholerae-Associated Necrotizing Fasciitis in an Immunocompromised Individual: Trivial Exposure Leading to a Life-Threatening Illness

**DOI:** 10.7759/cureus.69727

**Published:** 2024-09-19

**Authors:** Praveen Arumugam, Pankhuri Kumari, Naveen Kumar, Ashok Kumar, Ashok Kumar

**Affiliations:** 1 Internal Medicine, Max Smart Super Speciality Hospital, Saket, New Delhi, IND; 2 Microbiology, Max Smart Super Speciality Hospital, Saket, New Delhi, IND

**Keywords:** immunocompromised, laboratory risk indicator for necrotizing fasciitis (lrinec) score, necrotizing fasciitis, plhiv, skin blister, vibrio cholerae

## Abstract

*Vibrio cholerae* primarily causes gastrointestinal infections. However, in immunocompromised patients, the presentation can be atypical in the form of extraintestinal manifestation and more invasive disease. We report a patient with human immunodeficiency virus (HIV), who had necrotizing fasciitis in the left leg following exposure to stagnant rainwater. These immunocompromised patients should be educated about the possibility of such life-threatening infection following such a minor exposure.

## Introduction

Cholera, caused by *Vibrio cholerae*, is a waterborne disease. The natural history of cholera varies depending upon bacterial strains, inoculums, host genetics, and individual immune statuses. While most cases reported worldwide result in gastrointestinal illness, *V. cholerae* can occasionally cause extraintestinal infections, particularly in immunocompromised individuals, such as those with human immunodeficiency virus (HIV) [1]. This increases susceptibility to infections and results in a chronic carrier state. We report an interesting case of a male presenting with extensive and rapidly worsening cellulitis that did not improve with empirical antimicrobial therapy.

## Case presentation

**Figure 1:**
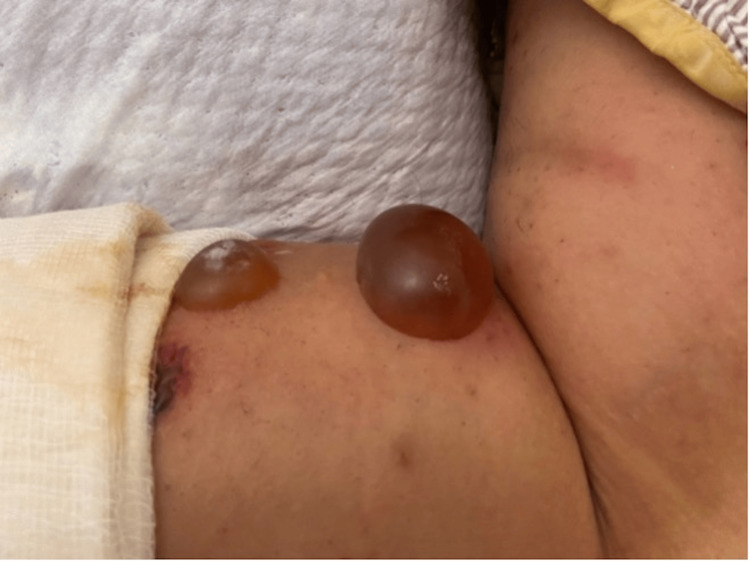
Cutaneous un-ruptured bullae in the left lower leg on arrival

**Figure 2:**
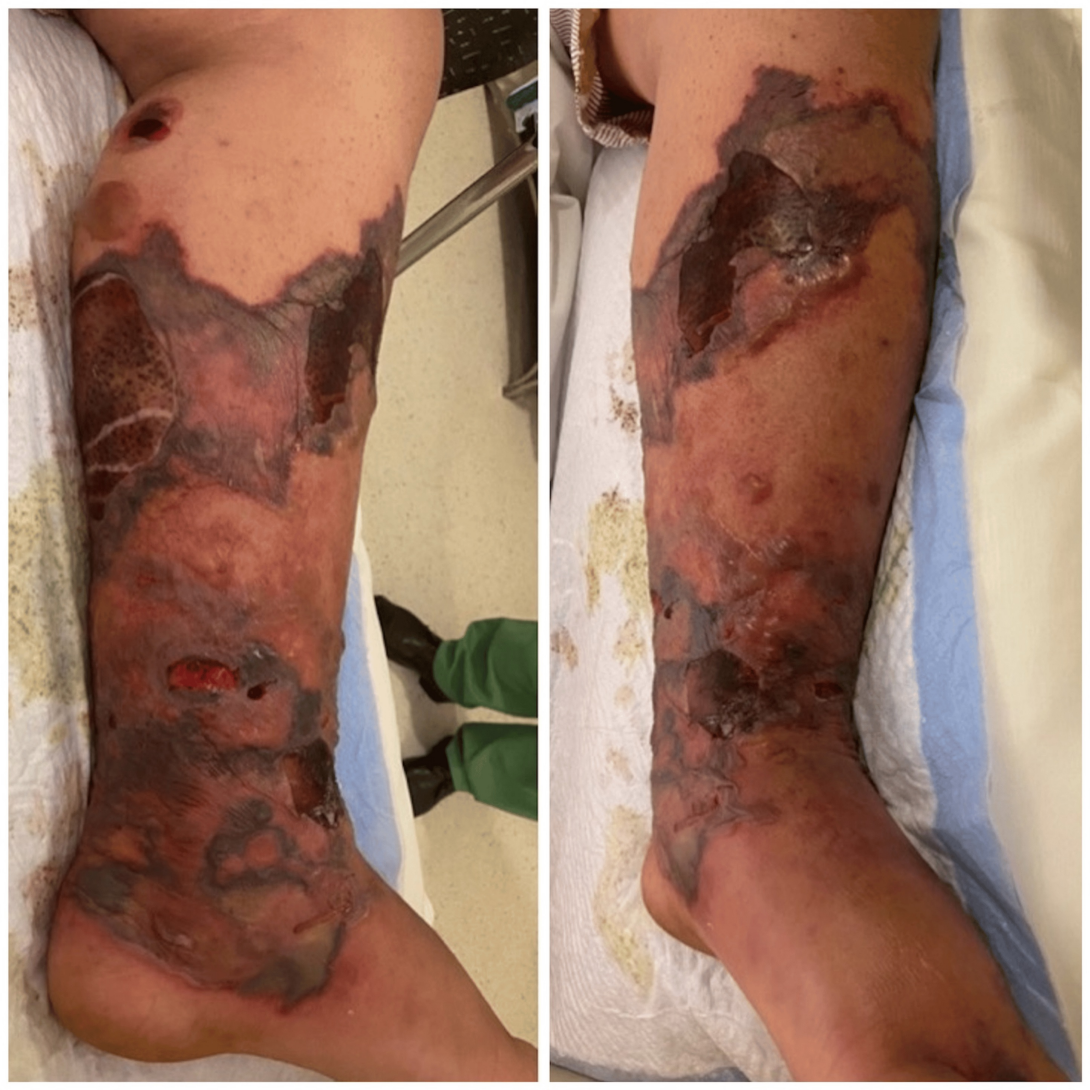
Ruptured multiple bullous lesions, along with discoloration of the skin suggestive of necrotizing fasciitis

A middle-aged male in his late 30s presented to the emergency department with swelling on his left leg that had progressed rapidly over the past day. He also noticed the development of blisters, which rapidly increased in size and number over the next 24 hours (Figure 1 and Figure 2). The swelling was accompanied by a dull, aching pain in his left leg, although he did not observe any discoloration in his toes. He revealed a history of walking through a waterlogged area a few days prior, during the rains. The patient had HIV disease, active hepatitis B, and autoimmune hepatitis (smooth muscle antibody-positive in 2016), for which he was only consuming entecavir. He had discontinued all other antiretroviral therapy (ART) medicines on his own a few months earlier. He denied any history of trauma or diabetes. On examination, the patient was obese, appeared toxic, and was acutely ill. He was febrile (temperature of 101°F), tachycardic (pulse rate of 130 beats per minute), and tachypneic (25 breaths per minute) and had a blood pressure of 150/80 mmHg and oxygen saturation of 95% at room temperature. His cardiac, respiratory, and abdominal examinations were normal. The skin over his left lower extremity, from the ankle to the knee, was erythematous, warm, swollen, and tense. Bullae were present, but no crepitus was observed. There was significant tenderness up to the mid-thigh area. However, pulses remained palpable in the lower limb. An emergency venous Doppler scan revealed subcutaneous edema in the lower left limb with no evidence of deep vein thrombosis. An ultrasonography of the abdomen showed changes associated with chronic liver disease and splenomegaly. Given the strong suspicion of necrotizing fasciitis (Laboratory Risk Indicator for Necrotizing Fasciitis (LRINEC) score = 7; an LRINEC score of ≥6 could be used as a potential tool to rule in necrotizing fasciitis, albeit a score <6 should not be used to rule out the diagnosis), he was counseled to undergo emergency incision and drainage. A multidisciplinary team was assembled for the procedure. However, the patient wanted to seek an opinion from a plastic surgeon at a government hospital, causing an unexpected delay. Although a magnetic resonance imaging (MRI) of the limb was suggested, it was not performed due to financial constraints.

Baseline investigations (Table 1) revealed abnormal liver and kidney functions, along with coagulopathy. C-reactive protein and D-dimer levels were evaluated. Fluid was aspirated from the blister and cultured to grow *V. cholerae*. Paired blood cultures obtained from both hands exhibited the growth of *V. cholerae*. Bacteria were sensitive to doxycycline, azithromycin, and ampicillin. Meanwhile, the patient was empirically treated with clindamycin and subsequently injected with doxycycline after receiving the sensitivity report. Following additional counseling and advice from an external doctor, he consented to the emergency procedure.

**Table 1:** Laboratory test results in serial order (right to left) AST/ALT: aspartate transaminase/alanine aminotransferase, PT/INR: prothrombin time (in seconds)/international normalized ratio

Parameters	Postoperative results	Results on the day of admission	Outside laboratory results	Reference ranges
Creatinine (mg/dL)	1.4	1.9	2.5	0.9-1.3
Urea (mg/dL)	129	123	101.9	17-43
Serum sodium (mmol/L)	128	126	122	136-146
Serum potassium (mmol/L)	3.54	4.16	3.92	3.5-5.1
Serum bicarbonate (mmol/L)	21	21	19	21-31
Serum calcium (mg/dL)	7.4	7.1		8.5-10.5
Serum albumin (g/dL)	-	1.9	-	3.5-4.5
Total protein (g/dL)	-	5.7	-	6.5-8
Direct bilirubin (mg/dL)	-	3.29	-	0-3
AST/ALT (IU/L)	82/85	110/72	-	15-41/17-63
PT/INR ratio	19.6/1.61	22.7/1.86	-	10.2-13.6
C-reactive protein	-	-	152.8	<5
D-dimer	-	-	1,472	0-243
CD4/CD8	-	-	297/211	30-61/12-42
Fibro scan	-	-	Stage 3 steatosis and cirrhosis of the liver	

An immediate consultation was conducted with a plastic surgeon, resulting in the patient undergoing pus drainage, debridement, and fasciotomy, with fresh frozen plasma being administered. Surgical findings revealed multiple pus pockets, along with necrosis of the skin and soft tissues, which were incised and cleaned. Meanwhile, an external examination showed that *V. cholerae* exhibited the non-O139 serotype. After initial stabilization, the patient chose to be discharged and managed at a government hospital due to financial constraints. The case was also reported to the public healthcare department.

## Discussion

*Vibrio cholerae* strains are globally distributed, particularly in regions adjacent to rivers and coastal regions, with cases peaking in warmer months [2]. While *V. cholerae* strains typically cause gastrointestinal illness after the consumption of contaminated food, severe invasive disease has been reported in immunocompromised patients [3-5]. A similar study by Ottaviani et al. reported on an HIV-positive patient with necrotizing fasciitis caused by *V. cholerae* O137 following a minor traumatic injury and exposure to seawater [1].

Necrotizing fasciitis, a life-threatening soft tissue infection, usually occurs in individuals with underlying chronic illness. While *Vibrio vulnificus* and non-cholera *Vibrio* species are well-recognized causes of necrotizing fasciitis, cases involving* V. cholerae* occur rarely [3,4]. In a case series of necrotizing fasciitis by Lee et al., *Vibrio* was found to be a causative agent in 36% of immunocompromised cirrhotic patients, for whom infections predominantly affected the lower limbs (70%) [3]. They observed that necrotizing fasciitis caused by gram-negative organisms was more commonly associated with bacteremia and septic shock. Our patient also exhibited lower limb involvement and bacteremia.

Apart from environmental factors and the specific *Vibrio* strain, host factors also appear to be crucial for the pathogenesis of infections caused by this group of microorganisms. In a case series identifying prognostic factors for fatality in invasive *Vibrio* infections, liver cirrhosis, malignancy, and steroid use were significantly associated with poor outcomes [4]. Early hospitalization and aggressive treatment were recommended for these patients, along with avoidance of exposure to such pathogens in high-risk individuals. Ko et al. reported various manifestations of *V. cholerae* (non-01 and non-0139) in cirrhotic patients, including septicemia with spontaneous bacterial peritonitis, soft tissue infections, acute gastrointestinal presentations, and necrotizing fasciitis or cellulitis from extremity wounds [6].

Ghosh et al. emphasized the role of proteolytic enzymes such as hemagglutinin protease, produced by *Vibrio cholerae* O1 in extraintestinal manifestations such as septicemia, wound infections, and hemorrhagic reactions [7]. Furthermore, Lin et al. presented a case of necrotizing fasciitis caused by non-O1/non-O139 *V. cholerae* in an individual with underlying chronic diseases such as diabetes, gout, and asthma following recent exposure to saltwater [8]. Tsuruta et al. also reported on the case of a Japanese woman who developed necrotizing fasciitis from non-01 and non-0139* V. cholerae*, leading to septic shock and subsequently requiring above-knee amputation after recovery from shock [9]. Despite being severely immunocompromised and receiving a timely diagnosis, the initial definitive management was delayed because the patient decided to seek an expert opinion. Fortunately, the outcome was favorable despite the involvement of a rare organism and the short delay in definitive management. Numerous case reports have described *V. cholerae* infections in HIV-positive individuals that often involved gastrointestinal symptoms or bloodstream sepsis following prior ingestion of seafood or exposure to seawater or freshwater [10-12]. Our case is unique as the pathogen was isolated from both tissue (presenting as cutaneous bullae) and blood, without any gastrointestinal manifestations.

## Conclusions

Considering the outlined risk factors, even minor skin exposure to a potential infected source can progress into a severe, life-threatening *V. cholerae* infection, especially in immunocompromised individuals. Public awareness and patient education programs can play a pivotal role in limiting the risk of exposure to these microorganisms among high-risk individuals. Early diagnosis and treatment are crucial to prevent further complications and improve outcomes. Hence, proper patient education and counseling are crucial in guiding informed decision-making.
